# Accumulation of Intraneuronal β-Amyloid 42 Peptides Is Associated with Early Changes in Microtubule-Associated Protein 2 in Neurites and Synapses

**DOI:** 10.1371/journal.pone.0051965

**Published:** 2013-01-23

**Authors:** Reisuke H. Takahashi, Estibaliz Capetillo-Zarate, Michael T. Lin, Teresa A. Milner, Gunnar K. Gouras

**Affiliations:** 1 Department of Anatomic Pathology, Tokyo Medical University, Tokyo, Japan; 2 Department of Neurology and Neuroscience, Weill Cornell Medical College, New York, New York, United States of America; 3 Laboratory of Neuroendocrinology, The Rockefeller University, New York, New York, United States of America; 4 Wallenberg Neuroscience Center, Department of Experimental Medical Science, Lund University, Lund, Sweden; Thomas Jefferson University, United States of America

## Abstract

Pathologic aggregation of β-amyloid (Aβ) peptide and the axonal microtubule-associated protein tau protein are hallmarks of Alzheimer's disease (AD). Evidence supports that Aβ peptide accumulation precedes microtubule-related pathology, although the link between Aβ and tau remains unclear. We previously provided evidence for early co-localization of Aβ42 peptides and hyperphosphorylated tau within postsynaptic terminals of CA1 dendrites in the hippocampus of AD transgenic mice. Here, we explore the relation between Aβ peptide accumulation and the dendritic, microtubule-associated protein 2 (MAP2) in the well-characterized amyloid precursor protein Swedish mutant transgenic mouse (Tg2576). We provide evidence that localized intraneuronal accumulation of Aβ42 peptides is spatially associated with reductions of MAP2 in dendrites and postsynaptic compartments of Tg2576 mice at early ages. Our data support that reduction in MAP2 begins at sites of Aβ42 monomer and low molecular weight oligomer (M/LMW) peptide accumulation. Cumulative evidence suggests that accumulation of M/LMW Aβ42 peptides occurs early, before high molecular weight oligomerization and plaque formation. Since synaptic alteration is the best pathologic correlate of cognitive dysfunction in AD, the spatial association of M/LMW Aβ peptide accumulation with pathology of MAP2 within neuronal processes and synaptic compartments early in the disease process reinforces the importance of intraneuronal Aβ accumulation in AD pathogenesis.

## Introduction

Alzheimer disease (AD) neuropathology is characterized by aggregation of the β-amyloid (Aβ) peptide in plaques and the hyperphosphorylated tau protein in neurofibrillary tangles (NFTs) [1]. Although AD plaques are extracellular Aβ aggregates, accumulation of Aβ42, the most pathogenic Aβ peptide, begins intraneuronally in AD [2]–[7] and transgenic AD mouse models [8]–[15]. In transgenic AD mice, cognitive impairments appear prior to plaques [16]–[19] accompanied by intraneuronal Aβ peptide accumulation [10], [18]–[20], suggesting that intraneuronal Aβ peptides are one of the earliest events of AD pathogenesis [21]. We previously demonstrated in brains of amyloid precursor protein (APP) Swedish mutant transgenic mice (Tg2576) that intraneuronal Aβ42 peptides accumulate with aging in endosomes, in particular multivesicular bodies (MVBs), in distal processes and synaptic compartments [13] prior to Aβ plaques. Moreover, prior to Aβ plaques, marked accumulation and oligomerization of Aβ42 peptides within processes and synaptic compartments was associated with subcellular pathology, including a reduced or absent microtubular network [13], [22]. Recently, we reported co-localization of Aβ42 and phosphorylated tau at synapses in areas without plaques [23]. It has been suggested that abnormally hyperphosphorylated tau inhibits assembly of and disrupts microtubules, resulting in sequestration of microtubule-associated proteins (MAPs) [24], [25]. The accumulation of Aβ peptides and associated structural and functional alterations in MAPs within synapses are significant, because synaptic alterations are the best pathologic correlate of cognitive dysfunction [26].

Increasing evidence suggests that soluble, low molecular weight (LMW) Aβ42 oligomers are pathogenic, although determination of which precise species may be most toxic in the brain is technically challenging and controversial. Levels of soluble Aβ42 peptides correlate better with synaptic loss and cognitive dysfunction than plaques [27]–[29]. The soluble Aβ fraction is composed primarily of Aβ monomers and SDS-stable LMW Aβ oligomers, specifically dimers and trimers [27]–[29]. Aβ dimers have been detected in the hippocampal CA1 region and entorhinal cortex of aging human brain even in the absence of amyloid plaques or NFTs [30]. Infusion of soluble Aβ dimers and trimers into rodent brain induced cognitive impairments [31]. These reports suggest that the accumulation of Aβ42, especially LMW Aβ42 oligomers, may be important pathogenically in AD.

Using the well-characterized anatomy of the hippocampus, we previously showed that Aβ42 accumulation within postsynaptic compartments of dendrites in CA1 neurons of the hippocampus was associated with early pathological redistribution and phosphorylation of tau [23]. In the present study, we set out to explore Aβ42 accumulation in dendrites compared to axons using MAP2 as a maker of dendrites and tau-1 as a marker for axons in Tg2576 mice. Yet, we noticed that as M/LMW Aβ42 peptides increased with aging in dendrites, including in postsynaptic compartments, levels of MAP2 decreased, eventually vanishing in the presence of high molecular weight (HMW) Aβ42 oligomers or thioflavin S positive β-pleated amyloid in mouse brains. Accumulation of M/LMW Aβ42 peptides also occurred, although to a lesser extent, in tau-1 positive axons. Our data support the scenario that localized subcellular accumulation of M/LMW Aβ42 peptides in dendrites is spatially associated with very early pathology of MAP2.

## Materials and Methods

### Mice

Well-established Tg2576 mice with the human APP Swedish 670/671 mutation were used in this study. APP knockout mice were generously provided by Dr. Hui Zheng, Baylor College of Medicine, Houston, TX. Brain sections from Tg2576 and wild-type mice were analyzed at 2–3, 9–11, 17–18, and 26 months of age with at least n = 3 for quantification experiments and n = 2–3 for immunolabeling studies.

All mouse experiments were performed in strict compliance with the institutional guidelines of the Institutional Animal Care and Use Committee (IACUC) of Weill Cornell Medical College, New York, NY, USA, in accordance with National Institutes of Health guidelines. The protocol was approved by the Weill Cornell IACUC (Protocol Number #03039-162A). All perfusions were performed under deep isoflurane anesthesia followed by decapitation. All efforts were made to minimize suffering.

### Antibodies

Aβ42 antibody AB5078P (Chemicon, Temecula, CA/Millipore, Billerica, MA) is a rabbit polyclonal antibody directed against the C-terminus of Aβ42 [32], [33]. This antibody had been biochemically characterized by Western blotting and mass spectrometry [32]. Antibody AB5078P was further reported to not cross-react to APP on Western blot by Agholme et al., [34]. In addition, we observed the same labeling pattern (predominately associated with MVBs and smaller vesicles but not with the Golgi apparatus) with this Aβ42 antibody AB5078P (Figure S1A) as we had with Aβ42 antibody MBC42, which we had characterized by Western blotting, immunohistochemistry and immuno-EM [13]. In contrast, immuno-EM labeling of APP indicates predominant localization to the Golgi apparatus [13], [35]. The AB5078P antibody recognizes monomers and to a lesser extent dimers and trimers (see Results). We note that Millipore ran out of this clone and that the currently available version of AB5078P is therefore not the same as the one we used in the present and prior studies, despite the use of the same clone number. M16 is a rabbit polyclonal antibody (kindly provided by Dr. Charles Glabe, University of California, Irvine, CA), raised against synthetic Aβ1–42; it preferentially recognizes insoluble HMW Aβ42 oligomers [13], [32], [33], [36], [37]. 6E10 (Signet Laboratories/COVANCE, Princeton, NJ) is a monoclonal antibody directed against residues 5–10 of Aβ peptides, and thus also recognizes full-length APP; it detects Aβ42 monomers and a wide variety of LMW and HMW Aβ42 oligomers [36]. Other antibodies used in this study: MAP2 (Sigma, St. Louis, MO) for dendrites, tau-1 (Chemicon/Millipore) for non-phosphorylated tau localized in axons.

### Tissue preparation

Preparation of tissue sections from Tg2576 mice [38] was similar to that described previously [13], [38]. Mice were anesthetized with sodium pentobarbital (150 mg/kg, i.p.) and perfused via the ascending aorta with saline/heparin followed by 40 ml of 3.75% acrolein (Polyscience, Warrington, PA), 2% paraformaldehyde in 0.1 M phosphate buffer (PB), pH 7.4. Tissue sections were cut on a vibratome (40 µm thick) and were kept in storage buffer composed of 30% sucrose and 30% ethylene glycol in PB at −20°C.

### Immunolabeling

Immunolabeling for light microscopy was performed as previously described [36], [39]. Free-floating sections were incubated in primary antibodies (MAP2 1∶1000, tau-1 1∶500) for 24 h at room temperature and then for 24–48 h at 4°C. The sections were incubated in biotinylated horse anti-mouse immunoglobulin (IgG) secondary antibody (1∶400, Vector Laboratories, Burlingame, CA) for 30 min, followed by the peroxidase-avidin complex (Vectastain ABC kit, Vector) for 30 min. The secondary antibody was diluted in 0.1 M Tris-saline (pH 7.6) containing 0.1% bovine serum albumin (BSA). The reaction product with the ABC kit was visualized after incubation of sections with 3, 3′-diaminobenzidine (Aldrich Chemical, Milwaukee, WI) and hydrogen peroxide. The sections were observed using a system consisting of a Nikon Eclipse E600 microscope (Morrell Instrument Co., Melville, NY) equipped with a computer-controlled LEP BioPoint motorized stage (Ludl Electronic Products, Hawthorne, NY), a DEI-750 video camera (Optronics, Goleta, CA), and a Dell Dimension 4300 computer (Dell, Round Rock, TX).

For immunofluorescence, free-floating sections were first incubated in primary antibodies (Aβ42 1∶200, M16 1∶1000, MAP2 1∶1000, tau-1 1∶500) for 24 h at room temperature and then for 24–48 h at 4°C, followed by appropriate fluorescent secondary antibodies Alexa 488 goat anti-rabbit IgG (green) and Alexa 546 goat anti-mouse IgG (red) (Molecular Probes, Eugene, OR) for 1 h at 37°C. Images were taken using an Olympus IX70 microscope with a Hamamatsu digital camera.

### Immunohistochemical analysis

For MAP2 quantification, MAP2 immuno-stained brain sections were viewed using a Nikon Eclipse E600 microscope with a 40× objective, and digital images were captured using the Stereo Investigator 4.35 software program (Microbrightfield, Williston, VT). Photomicrographs were prepared by adjusting levels, brightness and contrast in Adobe Photoshop 7.0. Images of areas from stratum radiatum (SR) of the hippocampal CA1 region were captured using NIH Image 1.63 (National Institutes of Health, Bethesda, MD). For quantitative densitometry, ten random areas from SR of the hippocampal CA1 region in Tg2576 and wild-type mice (*n* = 3, each) were analyzed. Immunoreactivity of MAP2 in the SR region was measured and indicated as arbitrary units with the mean gray value of 256 gray levels. To compensate for background staining between images, the average pixel density for 3 regions without any immunoreactivity on brain section was subtracted. Tissues from Tg2576 and wild-type mouse brain sections were processed together in the same crucibles. Optical density values were measured using NIH image. Net optical density values obtained after subtracting background values were indicated as arbitrary units.

Ratio of MAP2-immunoreactivity in arbitrary units in the SR region of Tg2576 mouse brains divided by that of wild-type mice were calculated at 2–3, 9–11, and 17–18 month-old mice. The ratio was standardized to 100% for MAP2-immunoreactivity arbitrary units of wild-type mice. A comparison was performed between 2–3 months and 9–11 months, 2–3 months and 17–18 months of age, respectively using Student's *t* test for statistical analysis. Data were expressed as the mean ±SEM, and the significance threshold was *p*<0.05.

### Immuno-electron microscopy

Immuno-electron microscopy (EM) localization was performed as previously described [13], [38]. Free-floating sections for dual-labeling immuno-EM were incubated with Aβ42 (AB5078P 1∶50) and MAP2 (1∶1000) antibody and processed first for immunoperoxidase localization of MAP2 antibody as described above. Sections then were processed by the immunogold-silver method for localization of Aβ42 antibody. For this, sections were incubated with goat anti-rabbit IgG conjugated to 1 nm gold particles (AuroProbe One; Amersham Biosciences, Arlington Heights, IL) in 0.01% gelatin and 0.08% BSA in PBS, pH 7.4, for 2 hours at room temperature. Sections were rinsed in PBS, post-fixed in 2% glutaraldehyde in PBS for 10 minutes, and rinsed in PBS and 0.2 M sodium citrate buffer (pH 7.4). Conjugated gold particles were enhanced by treatment with silver solution (Amersham Biosciences). Sections were fixed in 2% osmium tetroxide in PB, dehydrated, embedded in EMBed 812, sectioned (65- to 76-nm thick), and counterstained with uranyl acetate and Reynolds' lead citrate [39]. Final preparations were examined using a Philips CM10 electron microscope. Illustrations were generated from a high-resolution digital imaging CCD camera system (Advanced Microscopy Techniques, Danvers, MA) and processed using Adobe Photoshop 7.0 (Adobe System, Mountain View, CA).

Aβ42 and MAP2 immunolabeled profiles were classified as previously described by Peters et al. [40]. Somata were identified by the presence of a nucleus. Dendrites contained regular microtubule arrays and mitochondria, and were usually postsynaptic to axon terminal profiles. Axon terminals had numerous small synaptic vesicles, often contacting other neuronal profiles, and had a minimal diameter greater than 0.2 µm. Astrocytic profiles were recognized by their tendency to conform to the boundaries of surrounding profiles, by the presence of glial filaments and/or by less microtubules.

### Preparation and Western blotting of synthetic Aβ1–42 peptide*s*

Aβ1–42 was aggregated according to the method described by Fezoui et al., [41]. Briefly, lyophilized, synthetic Aβ1–42 peptides (American Peptide Company, Sunnyvale, CA) were dissolved in 20 mM NaOH, pH 10.5, to a final concentration of 1 mg/ml, sonicated, and lyophilized. Peptide was re-dissolved in water at a concentration of 1 mg/ml and filtered through a 0.22 µm Ultrafree-MC filter (Millipore, Bedford, MA). Aβ1–42 peptide (0.5 mg/ml) was buffered with 50 mM PB, 100 mM NaCl and incubated for 54 h at room temperature. We previously used this method to characterize the specificity of Aβ antibodies to different aggregated forms of Aβ1–42 peptides [36]; this method shows reproducible ranges of Aβ1–42 peptides from monomers to HMV oligomers on Western blot. Five hundred nanograms of aggregated Aβ1–42 peptides were separated by electrophoresis in 10–20% Tris-Tricine sodium dodecyl sulfate (SDS) polyacrylamide gels (Invitrogen, Carlsbad, CA), transferred to polyvinylidene-difluoride membranes (Millipore), and blotted with Aβ42 (1∶300) or 6E10 (1∶1000) antibodies, followed by incubation with secondary-HRP antibodies and visualization after enhanced chemiluminescence (Amersham Biosciences).

## Results

### Aβ42 antibody preferentially recognizes M/LMW Aβ42 peptides

**Figure 1:**
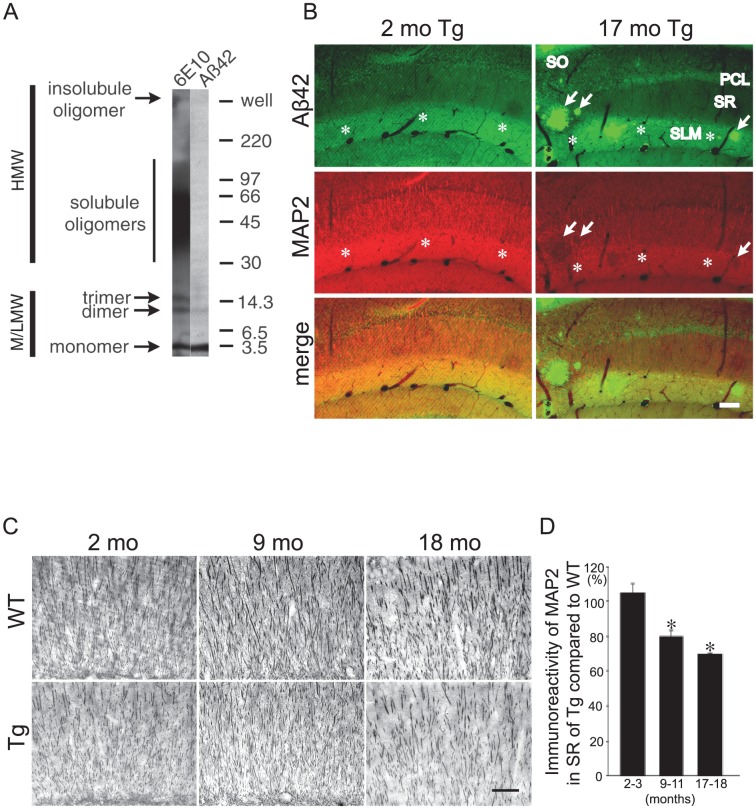
Aβ42 peptide accumulation and MAP2 reduction in Tg2576 mouse brains with aging. (A) Aggregation-state specificity of Aβ42 antibody (AB5078P) using gel electrophoresis and Western blot. The sample in the Western blot is *in vitro* aggregated synthetic Aβ1–42 polypeptide. The Aβ42 antibody prominently detected Aβ1–42 monomers and some low molecular weight oligomers (M/LMW) but not high molecular weight (HMW) oligomers (right lane). In contrast, Aβ antibody 6E10 detected M/LMW and HMW Aβ1–42 oligomers (left lane). (B) M/LMW Aβ42 peptides and MAP2 immunofluorescence in rostral CA1 hippocampal subregions. Tg2576 mouse brains were double labeled with antibodies against M/LMW Aβ42 (green) and MAP2 (red). Accumulation of M/LMW Aβ42 peptides correlated with reduced MAP2-immunoreactivity in CA1 with aging in Tg2576 mice between 2 and 17-months of age. MAP2 reduction was especially apparent in CA1 stratum lacunosum-moleculare (SLM, asterisks) and some reduction was also observed in the stratum radiatum (SR). MAP2 reduction was remarkable at the site where amyloid plaques were formed (arrows). Abbreviations: SO, stratum oriens; PCL, pyramidal cell layer; SR, stratum radiatum; SLM, stratum lacunosum-moleculare. Scale bar: 80 µm. (C) Representative images of the SR of the CA1 region of hippocampus demonstrated reduced MAP2 immunoperoxidase staining with aging (2, 9 and 18 months of age) in Tg2576 compared to wild-type mouse brains. Scale bar: 50 µm. (D) Quantification of MAP2-immunoreactivity in the SR of the hippocampal CA1 region revealed an age-related decrease in Tg2576 with aging at 9–11 and 17–18 months compared to at 2–3 months. Ratio of MAP2-immunoreactivity in the SR region of Tg2576 divided by that of wild-type mice were calculated at 2–3, 9–11, and 17–18 month-old mice, respectively. The ratio was standardized to 100% for MAP2-immunoreactivity of wild-type mouse. A comparison was performed between 2–3 months and 9–11 months, and 2–3 months and 17–18 months of age (Student's *t* test, * denotes significance: *p*<0.05).

We previously reported that immunoreactivity for the HMW Aβ42 oligomer-specific antibody M16 is absent in brains of wild-type mice at all ages, and in brains of Tg2576 mice until just prior to plaque formation, where it consistently was associated with structural pathology within neurites [36]. To characterize potential effects of Aβ42 peptide accumulation prior to HMW oligomerization, we sought an antibody that visualized M/LMW but not HMW Aβ42 oligomers. To analyze which conformations of Aβ42 peptides are recognized by the well-characterized Aβ42 antibody AB5078P [32], we immunoblotted synthetic Aβ1–42 peptides prepared to include monomers to oligomers [36]. By Western blotting, the Aβ42 antibody detected predominantly Aβ1–42 monomers (Figure 1A). Importantly, HMW Aβ1–42 oligomers were not detected. Modest band intensities of LMW Aβ1–42 dimers and trimers could be observed after longer exposure. In contrast, and as previously reported [36], [42], the Aβ domain/APP antibody 6E10 detected Aβ1–42 monomers and a wide variety of LMW and HMW oligomers (Figure 1A). We previously confirmed the specificity of the Aβ42 antibody (AB5078P) [43] by absence of immunofluorescence in cultured neurons derived from well-established APP knockout mice [44] compared to punctate/vesicular labeling in wild-type mice. Additionally, lack of appreciable labeling was seen in APP knockout mouse brains (Figure S1B). Despite these characterization studies, one cannot fully exclude that conformational specificity of Aβ antibodies may differ between Western blotting and immunohistochemistry.

### M/LMW Aβ42 peptide accumulation and MAP2 reduction in dendrites of Tg2576 mouse brains with aging

Biochemical analyses have shown that Aβ42 peptides increase with aging in brains of Tg2576 mice even prior to plaque formation [45]. By immuno-EM, Aβ42 peptide accumulation was evident especially within distal neuronal processes and synaptic compartments with aging rather than in cell bodies [13]. However, which conformation of Aβ42 peptide induces the earliest pathological alterations and how Aβ42 peptide accumulation relates to pathology of microtubule-associated proteins remains unclear. Utilizing the well-defined anatomy of the hippocampus, we show in Tg2576 mice early and progressive M/LMW Aβ42 peptide accumulation with aging, especially in the stratum lacunosum-moleculare (SLM) of the hippocampal CA1 region (Figure 1B, upper panel). This region contains predominantly distal dendrites from CA1 pyramidal cells and their associated synaptic compartments. We next investigated M/LMW Aβ42 peptide accumulation in relation to the dendritic microtubule-associated protein, MAP2, in the hippocampus of Tg2576 mice with aging. Remarkably, as the immunoreactivity for Aβ42 peptides increased, that for MAP2 decreased in CA1 dendrites of Tg2576 mice with aging. This was especially noticeable in the SLM (Figure 1B). We also observed some decrease in the stratum radiatum (SR). Co-localization of Aβ42 and MAP2 in the SR region is much clearer with higher magnifications, although it is not as clear as in the SLM or PCL (data not shown). On the whole, MAP2 reduction is marked in the SLM, and is not as clear in the SR region. To confirm and quantify the decrease in MAP2-immunoreactivity in Tg2576 mice, sections of Tg2576 and wild-type mouse brains at different ages were labeled with MAP2 antibody by immunoperoxidase. Dendrites in the SR of the CA1 region of aging Tg2576 mice appeared sparser and of reduced caliber compared to age-matched wild-type mice, which was especially evident at 17–18 months of age (Figure 1C). This is consistent with several previous studies demonstrating reduced dendritic architecture in APP mutant transgenic mice [46]–[48]. Densitometric quantification showed a significant decline in MAP2-immunoreactivity in the SR of the CA1 region of Tg2576 mice as a function of aging (Figure 1D). Specifically, MAP2-immunoreactivity in the SR of CA1 proximal dendrites was 105±5.08% (*n* = 3; p = 0.444), 79.64±3.23% (*n* = 3; p = 0.034) and 69.69±0.25% (*n* = 3; p<0.001) that of wild-type mice at 2–3 months, 9–11 months and 17–18 months of age, respectively. Similar reductions in MAP2-immunoreactivity in Tg2576 compared to wild-type mouse hippocampi were also apparent in the region of CA1 terminal dendrites in the SLM (Figure S2). In contrast to the MAP2 reductions, we did not detect any appreciable changes in tubulin and actin (data not shown).

### Ultrastructural analysis of M/LMW Aβ42 peptides and MAP2

**Figure 2:**
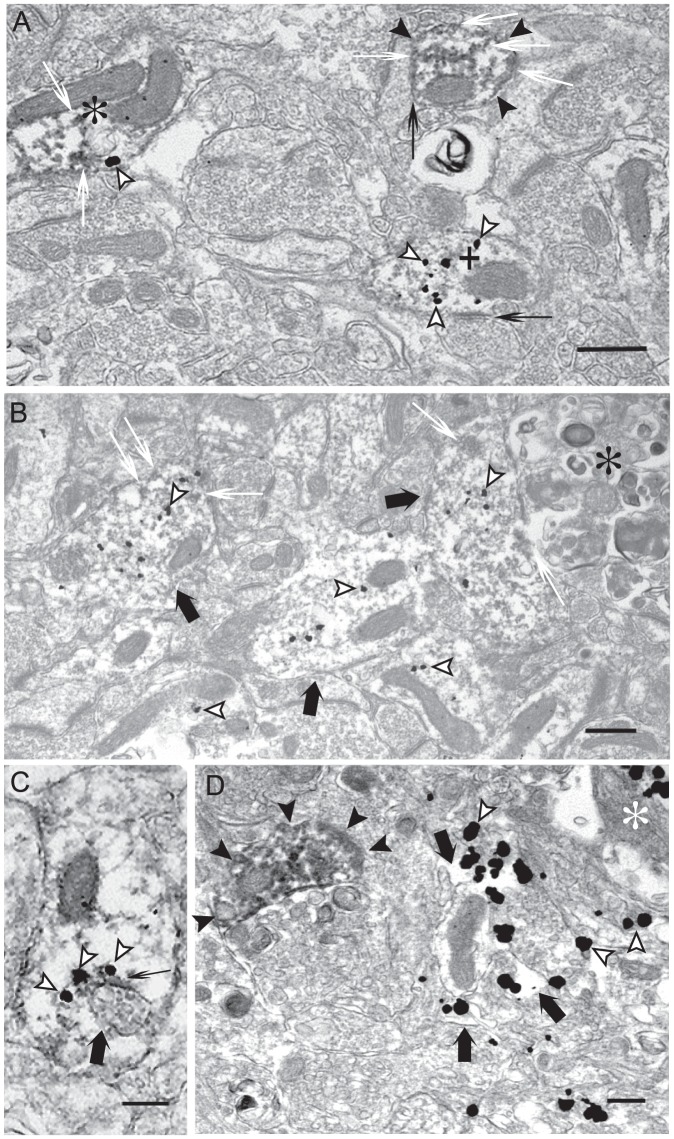
Accumulation of M/LMW Aβ42 peptides in dendrites correlates with reduced MAP2 in Tg2576 mouse brain by immuno-EM. (A) Representative image from an area not adjacent to amyloid plaques in a 26-month-old Tg2576 mouse. Marked M/LMW Aβ42 peptide (gold particles, white arrowheads) accumulation was locally associated with reduced MAP2 (immunoperoxidase, white thin arrows) staining in a postsynaptic compartment (plus), while a strongly MAP2 labeled dendritic postsynaptic compartment revealed no Aβ42 labeling (black arrowheads). Moreover, another synaptic compartment revealed intermediate levels of both M/LMW Aβ42- and MAP2-immunoreactivities (asterisk). Synaptic compartments are clearly recognizable because of the presence of postsynaptic densities (black thin arrows). (B) Adjacent to dystrophic neurites (asterisk), M/LMW Aβ42 peptide (gold particles, white arrowheads) accumulation is apparent in dendrites (thick black arrows) with reduced labeling of MAP2 (immunoperoxidase, white thin arrows). (C) Aβ42 peptide (gold particles, white arrowheads) associated with dark fibril-like material (black thin arrow) attach to an abnormal appearing multivesicular body (thick black arrow) in a weak MAP2-labeled dendrite. (D) Marked accumulation of Aβ42-immunoreactivity (gold particles, white arrowheads) in degenerated pre- and postsynaptic compartments without any obvious cytoskeletal structure (thick black arrows) close to amyloid fibers (asterisk). In contrast, a normal MAP2-labeled postsynaptic compartment without M/LMW Aβ42 peptide labeling is located nearby (black arrowheads). Scale bars: 500 nm.

Dual-labeling immuno-EM was used to analyze the subcellular pattern of M/LMW Aβ42 peptides and MAP2 in dendrites. In agreement with light microscopic results, we observed a correlation between increasing M/LMW Aβ42 peptide accumulation and reduced MAP2-immunoreactivity using EM (Figure 2). Postsynaptic compartments with no appreciable M/LMW Aβ42 peptide labeling showed strong MAP2 labeling; those with intermediate M/LMW Aβ42 peptide labeling showed intermediate MAP2 labeling; and those with strong M/LMW Aβ42 peptide labeling showed little MAP2 labeling (Figure 2A). Such images support that reduced MAP2 is not merely secondary to loss of dendrites, since less MAP2 labeling is seen in these still present M/LMW Aβ42 labeling dendrites and postsynaptic compartments. Near or adjacent to clearly dystrophic neurites, numerous M/LMW Aβ42 gold particles could be seen in dendrites and postsynaptic compartments that contained only slight MAP2-immunoreactivity (Figure 2B). At times, M/LMW Aβ42 gold particles were found to be directly associated with degenerated multivesicular bodies (MVBs) near fibril-like electron-dense material, which could represent early Aβ42 fibrils (Figure 2C, black thin arrow). We also observed marked accumulations of M/LMW Aβ42 peptides in degenerated dendrites or axon terminals lacking any clear cytoskeletal architecture (Figure 2D, black arrows). At the same time, normal appearing dendrites and postsynaptic compartments with strong immunoreactivity for MAP2 and little intracellular Aβ42 could occasionally be found even near to extracellular amyloid fibers representing an amyloid plaque (Figure 2D).

### Lack of MAP2 at sites of HMW Aβ42 oligomers within dendrites

**Figure 3:**
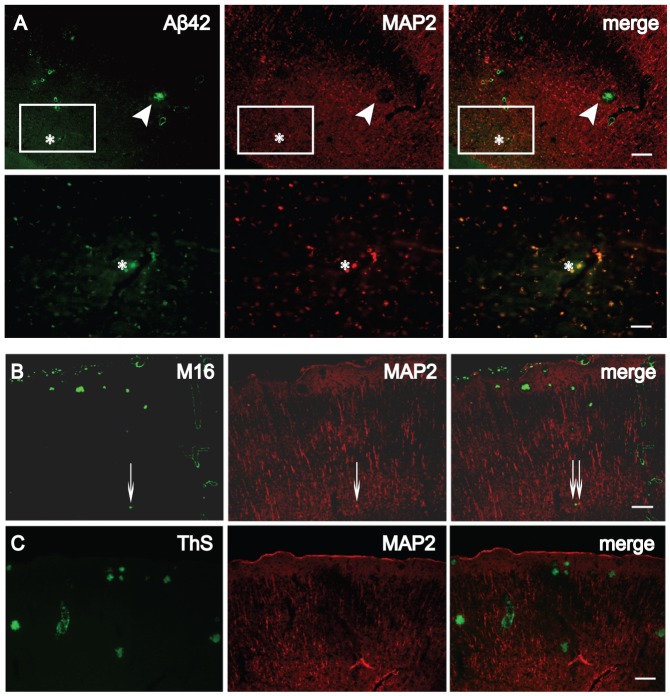
Lack of co-localization of HMW Aβ42 oligomers or thioflavin S-positive plaques with MAP2. (A) 26-month-old Tg2576 brain sections stained with Aβ42 (green) and MAP2 (red) antibodies. Top: MAP2-immunoreactivity was absent around an Aβ42 plaque (arrowhead). Bottom: A large MAP2-positive dendrite (∼56 µm^2^, asterisk) with M/LMW Aβ42 peptide accumulation in a higher magnification image of the rectangle in the top panel. (B) In contrast to M/LMW Aβ42 peptides, HMW Aβ42 oligomers detected by M16 antibody did not co-localize with MAP2, even in very small HMW Aβ42 oligomer accumulations (thin arrows). (C) Thioflavin S (ThS) staining for β-pleated Aβ fibrils never revealed co-localization with MAP2. Scale bars, 80 µm (A, top); 20 µm (A, bottom); 100 µm (B and C).

Figures 1 and 2 indicated that dendritic accumulation of M/LMW Aβ42 peptide is locally associated with decreases in MAP2. Co-localization of reducing MAP2 and M/LMW Aβ42 still occurs (Figure 1B merge, Figure 2A asterisk), particularly within large puncta in SR and stratum oriens (SO), which could represent focal sites of swollen, dystrophic neurites (Figure 3A, bottom). In contrast, there was no labeling of MAP2 within Aβ-labeled plaques or in a halo around plaques (Figure 3A, top). Since dendritic MAP2-immunoreactivity was reduced in the presence of M/LMW Aβ42 oligomers (an early stage of Aβ aggregation) and absent in plaques (a later stage of Aβ aggregation), we next investigated the relationship of MAP2 with HMW Aβ42 oligomers (an intermediate stage of Aβ aggregation) using the well-characterized antibody M16 [37]. We previously confirmed by Western blotting that M16 reacts preferentially with HMW Aβ1–42 oligomers and characterized the time course of M16- immunoreactivity in Tg2576 mice at different ages [36]. Immunoreactivity for M16 did not appear until just before plaques and by immuno-EM was invariably associated with localized subcellular pathology [36]. We now show that in neurites of Tg2576 brain sections co-stained with M16 and MAP2 antibodies, there was no co-localization of MAP2 with HMW Aβ42 oligomers (Figure 3B). There was no overlap of MAP2- immunoreactivity with thioflavin S staining, which detects β-pleated amyloid fibrils, either (Figure 3C). These data support that accumulation of early stage Aβ aggregates (M/LMW Aβ oligomers) is already locally associated with progressive cytoskeletal pathology, which is severe when later stage aggregates (HMW Aβ oligomers and Aβ fibrils) are present.

### M/LMW Aβ42 peptides accumulate less in axons than in dendrites of Tg2576 mouse brains

Our data indicated that M/LMW Aβ42 peptide accumulation was associated with early alterations in the microtubule-associated protein MAP2 in dendrites. A hallmark of AD is the abnormal hyperphosphorylation of the microtubule-associated protein tau; unphosphorylated tau is also used as a marker for axons. We therefore next analyzed M/LMW Aβ42 oligomer and tau-immunoreactivities in Tg2576 mouse brains at 17–18 months, and 26 months of age. At 17–18 months, when M/LMW Aβ42-immunoreactivity accumulated markedly in dendrites, there was only modest M/LMW Aβ42 peptide co-localization with tau-1 antibody (specific for non-phosphrylated tau localized only in axons) in axons compared to dendrites (Figure S3, top, Figure 1B, bottom). In contrast, M/LMW Aβ42 peptide and tau-1 co-localization was less evident in wild-type mice (Figure S3, middle). In very old Tg2576 mice (26 months), with more marked M/LMW Aβ42 peptide accumulations, co-localization of M/LMW Aβ42 peptide and tau-1 became more evident (Figure S3, bottom), but not as apparent as it is for MAP2 (Figure 3A, bottom). These results indicate that M/LMW Aβ42 peptides accumulate with aging also in axons of Tg2576 mouse brains, although to a lesser extent than in dendrites. These results are consistent with our initial immuno-EM studies of Aβ42 peptide accumulation, which had been noted to occur in axons and dendrites, including their pre- and postsynaptic compartments, although it was most obvious within distal dendrites [13].

## Discussion

The relationship between Aβ peptides and the axonal protein tau, and how they are involved in pathology, are central questions in AD research. We previously provided immuno-EM data linking early tau hyperphosphorylation with intraneuronal Aβ peptide accumulation [23]. We showed that at older ages hyperphosphorylated tau co-localized with accumulating Aβ42 peptides within dystrophic neurites around plaques in Tg2576 mice. In 3×Tg mice aberrant accumulation of Aβ and phosphorylation of tau occurred early and prominently in distal dendrites and postsynaptic compartments. Moreover, we provided ultrastructural evidence of paired helical filaments (PHFs) in the 3×Tg mice [23], [49]. We noticed that Aβ progressively and prominently accumulates with aging in distal neuronal processes, and therefore for the current study set out to explore the accumulation of Aβ42 in relation to dendrites and axons in a well-established transgenic AD mouse model [13]. We used MAP2 as a dendritic marker and tau-1 as an axonal marker.

We now demonstrate that Aβ42 peptides accumulate prominently in dendrites, and that dendritic accumulation of Aβ42 peptides is locally associated with progressive reduction in MAP2. This reduction in MAP2 is consistent with previous studies documenting early dendritic degeneration and reduction in total dendritic area in transgenic AD mouse models [46]–[48], [50], [51]. However, the reduction in MAP2 is not solely due to loss of dendrites, because ultrastructurally still-existing dendrites and postsynaptic compartments showed reduced MAP2 staining with Aβ42 peptide accumulations in the same compartment. Our results also advise caution in using MAP2 as a marker of dendrites in trying to define dystrophic neurites as axonal or dendritic in human AD and AD transgenic models.

Synaptic alterations are the best correlate of cognitive dysfunction in AD [52], [53]. Although plaques and tangles are not obviously linked to synapses, our work shows that Aβ, tau and also MAP2 abnormalities occur at distal neurites and synapses. Taking advantage of the well-defined anatomy of the hippocampus, we found that Aβ accumulation and MAP2 reduction occurred earliest and most prominently in the stratum lacunosum-moleculare (SLM), which contains the distal synaptic field of apical dendrites of CA1 pyramidal neurons.

Previous studies suggest that soluble Aβ oligomers represent the most toxic form of Aβ peptide and are critical in inducing cognitive impairment and synaptic dysfunction [28], [29], [54], [55]. In transgenic mutant APP mice, progressive learning deficits and impaired synaptic plasticity occurred concomitantly with decreases in immunostaining for synaptophysin [56] and MAP2 [50], [51], [57]. Since these deficits preceded plaque formation, these results support a role of soluble Aβ species in synaptic dysfunction.

We observed that accumulation of M/LMW Aβ42 peptides, an early stage of Aβ aggregation, is locally associated with decreases in MAP2, which vanishes in the presence of HMW Aβ42 oligomers or Aβ fibrils, later stages of Aβ aggregation. Of note, thioflavin S staining was reported within neurons and neurites of some transgenic mice [9], [22]. Our observations are consistent with previous studies emphasizing the importance of soluble Aβ species in initiating pathology. HMW Aβ42 oligomers, as well as extracellular amyloid plaques, likely also play a pathogenic role, although they appear following the initiation of MAP2 pathology. The determination of the exact conformation(s) of pathogenic Aβ42 in brain remains challenging, since the analytic methods used can lead to conformational changes. Newer non-denaturing biochemical methods will be required to better pinpoint the precise Aβ conformations important in the AD brain [58]–[60].

**Figure 4:**
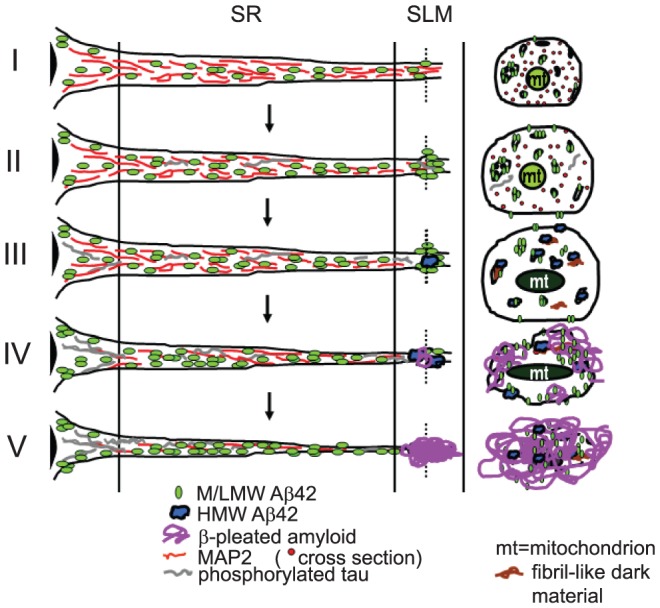
Schematic diagram of proposed sequence of Aβ42, MAP2 and tau alterations in a CA1 pyramidal cell apical dendrite with aging. At top (I), normal dendrite contains MAP2 associated with microtubules and M/LMW Aβ42 peptides. Cross section taken through the distal apical dendrite (stratum lacunosum-moleculare, SLM) is shown at right. (II) With aging, M/LMW Aβ42 peptides accumulate, which coincides with early reductions in MAP2, especially in the SLM. Later on in Tg2576 mice, Aβ42 M/LMW peptides co-localize with hyperphosphorylated tau in distal processes and synaptic compartments; this co-localization is more prominent in 3×Tg mice (23). (III) Subsequently, Aβ42 HMW oligomers develop in the distal dendrite, which is associated with localized absence of MAP2. Concomitantly, Aβ42 M/LMW peptides further accumulate in more proximal regions of the dendrite. (IV) This is followed by Aβ fibril formation, especially in distal neurites of the SLM and (V) deposition of amyloid plaques in SLM.

Based on these data, we propose a pathogenetic sequence linking intraneuronal Aβ42 accumulation with MAP2 pathologies and plaque formation (Figure 4). Our cumulative EM studies support the scenario that Aβ peptide deposition begins intraneuronally at synapses, leading then in the case of dendrites to loss of MAP2, followed finally by neuritic degeneration to initiate extracellular plaque formation. Our present data and schematic Figure 4 emphasize pathology in the apical dendrite terminal fields of the SLM; we focus on this area because of its well-defined anatomy. Synapses with intraneuronal Aβ accumulation are also located at other sites, consistent with location of plaques at other sites. More comprehensive studies are needed to better define the anatomic selectivity and relative predisposition of Aβ accumulation and associated pathology within dendrites versus axons in the AD brain.

We show that intraneuronal Aβ peptide accumulation is spatially associated with pathology of MAP2, providing a link between Aβ42 accumulation and MAP2 reduction. Aβ and MAP2 abnormalities are already prominent in synapses at early time points, a key observation given the importance of synaptic degeneration in cognitive dysfunction in AD. Finally, our data provide further support for the importance of M/LMW Aβ42 peptides in AD pathogenesis.

## Supporting Information

Figure S1 (A) Immuno-EM showing gold-particles of Aβ42 (using antibody AB5078P) are associated with the outer membrane of a MVB (arrows) close to the Golgi apparatus in an 11-month-old wild-type mouse brain, while no gold-particles are evident in the Golgi apparatus, in which full-length APP and APP CTFs (C-terminal fragments) are known to primarily reside. Abbreviations: MVB, multivesicular body; Golgi, Golgi apparatus; ER, endoplasmic reticulum; mit, mitochondrion; Endo, endosome; N, nucleus. Scale bars: 500 nm. (B) Lack of Aβ42-immunoreactivity in APP knockout mouse cortex with the Aβ42 antibody (AB5078P). Aβ42 is especially prominent in pyramidal neurons of layers IV and V of the wild-type mouse. Since Aβ42 peptides are generated by cleavage from APP, lack of Aβ42 labeling in 12-month-old APP knockout mouse brain is an important control. Abbreviations: WT, wild-type mouse; APPKO, APP knockout mouse. Scale bar: 100 µm (left column), 50 µm (right column).
(EPS)

Figure S2 MAP2 reduction in stratum lacunosum-moleculare (SLM) by immunoperoxidase labeling. Immunoreactivity for MAP2 was reduced in CA1 SLM of these representative 18-month-old Tg2576 (18 mo Tg) mice compared to age-matched wild-type (18 mo WT) mice (n = 3). Scale bar: 50 µm.
(EPS)

Figure S3 Accumulation of M/LMW Aβ42 peptides in tau-1-positive axons. (A) Immunofluorescent labeling of Tg2576 (top) and wild-type (bottom) mouse cortices revealed little M/LMW Aβ42 peptide co-localization (arrowheads) with tau-1-positive axons at 17 months of age, which was slightly more apparent in Tg2576 mice (n = 3). Bar: 20 µm. (B) More Aβ42 peptide accumulation in tau-1-positive axons was evident in very old (26 months) Tg2576 mouse brains; this is especially evident in enlarged axon puncta (arrowheads). Scale bar: 20 µm.
(EPS)
